# Visualizing membrane trafficking through the electron microscope: cryo-tomography of coat complexes

**DOI:** 10.1107/S2059798319005011

**Published:** 2019-04-30

**Authors:** Evgenia A. Markova, Giulia Zanetti

**Affiliations:** a Institute of Structural and Molecular Biology, Birkbeck College, Malet Street, London WC1E 7HX, England

**Keywords:** cryo-electron tomography, three-dimensional reconstruction, coat proteins, vesicular transport, COPII, subtomogram averaging

## Abstract

Coat proteins mediate vesicular transport between intracellular compartments, which is essential for the distribution of molecules within the eukaryotic cell. The global arrangement of coat proteins on the membrane is key to their function, and cryo-electron tomography and subtomogram averaging have been used to study membrane-bound coat proteins, providing crucial structural insight. This review outlines a workflow for the structural elucidation of coat proteins, incorporating recent developments in the collection and processing of cryo-electron tomography data. Recent work on coat protein I, coat protein II and retromer performed on *in vitro* reconstitutions or *in situ* is summarized. These studies have answered long-standing questions regarding the mechanisms of membrane binding, polymerization and assembly regulation of coat proteins.

## Abstract 2

The study of coat protein assemblies on membranes by cryo-electron tomography and subtomogram averaging is discussed, outlining the methodological advancements that enabled the gain of crucial structural insight.

## 1. Introduction   

Recent advances in cryo-electron microscopy (cryo-EM) have enabled numerous high-resolution studies which have addressed long-standing structural biology questions. The large body of cryo-EM work carried out for protein structure characterization has utilized single-particle electron microscopy (Cheng, 2018 ▸). Recently, developments in hardware, data collection and data processing have placed cryo-electron tomography (cryo-ET) and subtomogram averaging (STA) at the forefront of structural studies of repetitive assemblies, reaching resolutions comparable to those of single-particle EM (Schur *et al.*, 2016 ▸; Wan *et al.*, 2017 ▸; Hutchings *et al.*, 2018 ▸; Dodonova *et al.*, 2017 ▸; Himes & Zhang, 2018 ▸). This review discusses the application of cryo-ET to the study of intra­cellular coat proteins.

Eukaryotic cells are separated into membrane-bound compartments that constantly exchange molecules while retaining their own biochemical identity. This tightly controlled intercompartmental exchange is enabled by vesicular transport, in which coat proteins promote the formation of cargo-carrying vesicles from a donor compartment, and the budded vesicles travel towards a target compartment and fuse with it, delivering their cargo (Caro & Palade, 1964 ▸). Cargo molecules are selectively incorporated into vesicles by core coat proteins or additional interacting factors (Bonifacino & Glick, 2004 ▸). Different coat protein families are responsible for the transport between specific intracellular compartments, mediating local vesicular trafficking (Barlowe *et al.*, 1994 ▸).

**Figure 1:**
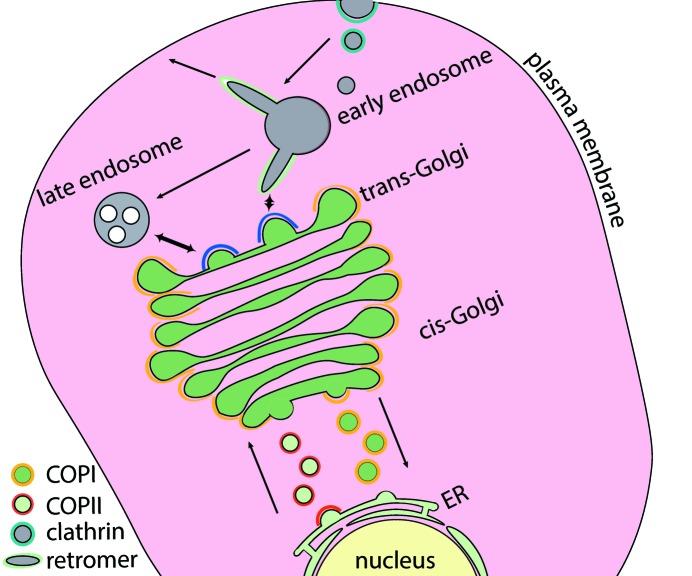
The intracellular function of coat proteins. Coat proteins are responsible for the exchange of biomolecules between membrane-bound compartments and facilitate endocytosis and exocytosis. COPII vesicles transport cargo from the endoplasmic reticulum (ER) to the Golgi apparatus (red), while COPI vesicles are responsible for intra-Golgi transport and trafficking from the Golgi to the ER (yellow). Retromer enables the tubulation of the early endosome towards the trans-Golgi network (TGN) and the cell exterior (green). Clathrin coats endocytic vesicles, which are formed at the plasma membrane, and buds emerging from the trans-Golgi network (blue).

**Figure 2:**
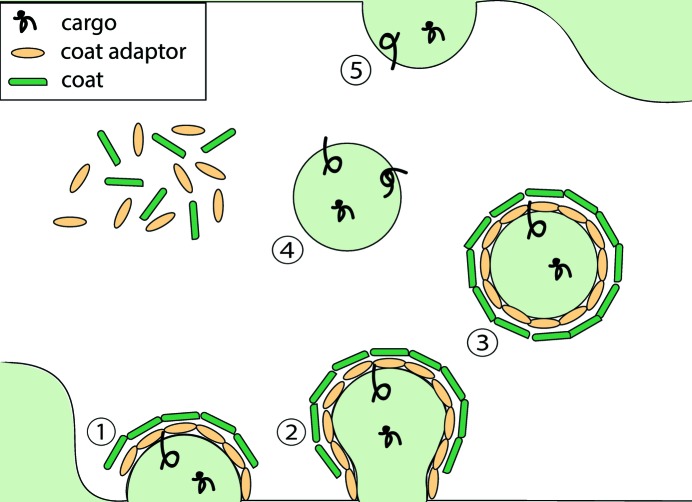
Vesicle budding and fusion. (1) Coat proteins are recruited to donor membranes, which have a characteristic lipid and protein composition and local curvature. Typically, a membrane-and-cargo-adapter-like layer and a coat-like membrane layer are assembled. Coat proteins and additional components concentrate cargo in the forming bud. (2) Membrane curvature increases and a vesicle neck forms. (3) Vesicle scission results in the release of the cargo-laden vesicle from the donor compartment. (4) Vesicle uncoating occurs, allowing subsequent fusion with the acceptor compartment. Coat proteins are released for recycling. (5) Fusion with the recipient compartment occurs and the release of cargo.

Four coat protein families have been described: coat protein II (COPII), coat protein I (COPI), clathrin and retromer (Fig. 1 ▸). The cytoplasmic coat protein complexes I and II (COPI and COPII) mediate retrograde and anterograde trafficking, respectively, between the endoplasmic reticulum (ER) and the Golgi apparatus (Orci *et al.*, 1986 ▸; Barlowe *et al.*, 1994 ▸). COPI is also responsible for trafficking between Golgi cisternae (Ishii *et al.*, 2016 ▸). Clathrin-coated vesicles mediate the transport of endocytic cargo from the plasma membrane, as well as trafficking from the trans-Golgi network to endosomal compartments (Roth & Porter, 1964 ▸; Robinson, 1994 ▸). Retromer mediates trafficking from the endosome to the trans-Golgi network and the plasma membrane (Seaman *et al.*, 1997 ▸; Temkin *et al.*, 2011 ▸). The common steps in vesicle formation shared by the known coat protein families are represented schematically in Fig. 2 ▸ (Bonifacino & Glick, 2004 ▸).

Questions that remain unanswered about coat proteins address the mechanism of their association with the membrane and their global assembly. Cryo-ET is well suited to address these questions as it can allow the high-resolution analysis of coat proteins in the process of modifying membranes.

## 2. A cryo-electron tomography workflow   

**Figure 3:**
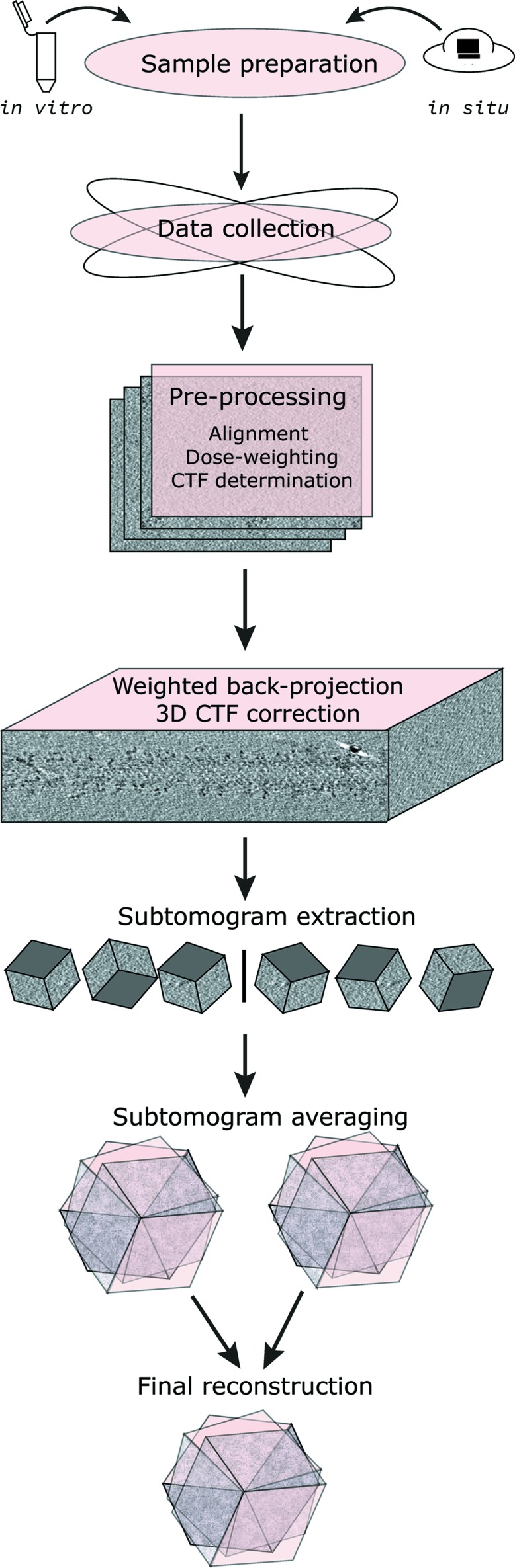
A cryo-electron tomography workflow used for the structural determination of COPII assembled on membranes.

We describe our workflow for the investigation of coat protein membrane assemblies by cryo-ET in Fig. 3 ▸.

### 2.1. Sample preparation   

Structures of isolated coat components and subcomplexes have been elucidated by X-ray crystallography and single-particle electron microscopy (Fotin *et al.*, 2004 ▸; Stagg *et al.*, 2006 ▸, 2008 ▸; Lee & Goldberg, 2010 ▸; Bi *et al.*, 2002 ▸, 2007 ▸; Mancias & Goldberg, 2008 ▸; Fath *et al.*, 2007 ▸; Wilbur *et al.*, 2010 ▸; Heldwein *et al.*, 2004 ▸; Collins *et al.*, 2002 ▸). However, isolated structures lack insight into the global interactions and arrangement defining the functional coats as assembled on membranes. This gap can be addressed by *in vitro* reconstitution studies with purified components assembled on artificial membranes (Dodonova *et al.*, 2017 ▸; Hutchings *et al.*, 2018 ▸; Kovtun *et al.*, 2018 ▸; Faini *et al.*, 2012 ▸; Bacia *et al.*, 2011 ▸). Alternatively, coated vesicles can be purified from cells (Cheng *et al.*, 2007 ▸; Malhotra *et al.*, 1989 ▸; Heymann *et al.*, 2013 ▸) or observed directly within cells (Bykov *et al.*, 2017 ▸; Kovtun *et al.*, 2018 ▸) for structural analysis. Cryo-ET and STA are then used to analyse the architecture of assembled coats, providing insights into the coat structure and its pleiomorphic assemblies (Zanetti *et al.*, 2013 ▸; Hutchings *et al.*, 2018 ▸; Kovtun *et al.*, 2018 ▸; Dodonova *et al.*, 2017 ▸, 2015 ▸; Faini *et al.*, 2012 ▸).

**Figure 4:**
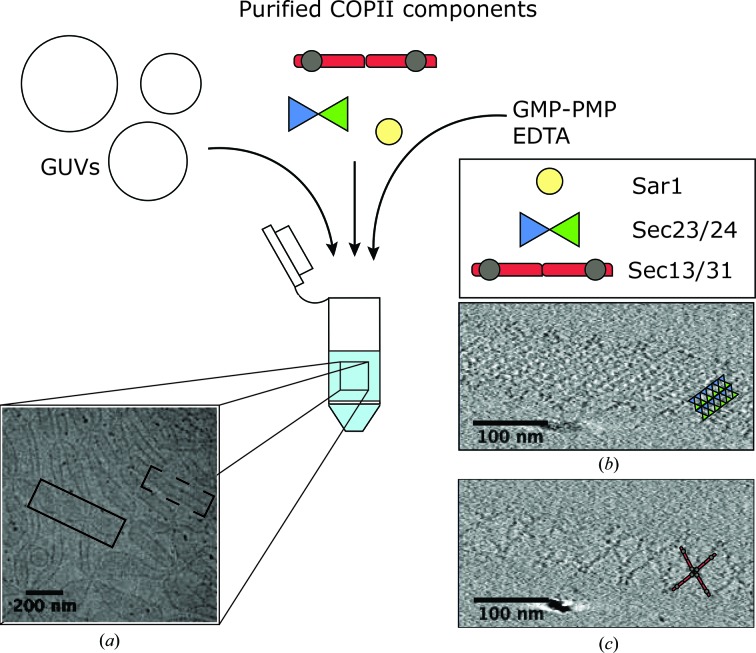
*In vitro* reconstituted COPII assemblies on membranes. The addition of purified COPII components to giant unilamellar vesicles results in COPII membrane binding and the generation of a variety of morphologies, including beads-on-a-string (dashed line) and tubular (unbroken line) COPII assemblies. (*a*) COPII morphologies seen in a medium-magnification image of a cryo-grid. (*b*, *c*) Slices through binned 8× cryo-tomograms with 50 iterations of SIRT-like filtering applied, showing the regular lattice assembly of inner (*b*) and outer (*c*) coat components. Tomogram images are courtesy of Joshua Hutchings (unpublished work).

As an example, we briefly describe the basic steps for COPII budding reconstitution *in vitro* (Fig. 4 ▸). The sequential assembly of the COPII coat components is well understood in yeast and is conserved in mammals. In the vesicle formation process, COPII proteins detect and induce membrane curvature, recognize and concentrate cargo, and bud the membrane into cargo carriers. COPII assembly is initiated by the recruitment of the Sar1 GTPase to ER exit sites (ERES). Sar1 associates with the membrane upon GTP binding, facilitated by its cognate guanine nucleotide-exchange factor (GEF), Sec12. GTP binding triggers a conformational change in Sar1, which inserts an ampiphatic N-terminal helix into the membrane. Sar1 then recruits Sec23/24, cytoplasmic heterodimers that form the COPII inner coat, which in turn recruit Sec13/31 heterodimers that assemble into the outer coat. This detaches the COPII vesicle, completing the COPII coat assembly, which is short-lived owing to an intrinsic propensity for disassembly. GTP hydrolysis by Sar1 is stimulated by the specific GAP activity of Sec23/24, and in turn accelerated by Sec13/31, resulting in dissociation of the COPII coat (Zanetti *et al.*, 2012 ▸; Bi *et al.*, 2007 ▸; Antonny *et al.*, 2001 ▸).

COPII budding has been reconstituted *in vitro* by incubating purified yeast COPII proteins with giant unilamellar vesicles (GUVs; Bacia *et al.*, 2011 ▸). The GUV composition and protein concentrations have been optimized to mimic physiological conditions and maximize budding efficiency (Matsuoka *et al.*, 1998 ▸; Bacia *et al.*, 2011 ▸). To stabilize the intrinsically unstable COPII complexes on the membrane, GMP-PNP, a nonhydrolysable GTP analogue, must be used (Bi *et al.*, 2007 ▸; Bacia *et al.*, 2011 ▸). Gold fiducial markers are added to reconstituted reactions prior to plunge-freezing to facilitate cryo-tomography tilt series alignment. Reconstituted COPII ‘budding’ reactions produce a variety of COPII morphologies, including budded and straight membrane tubules coated with COPII (Fig. 4 ▸).

### 2.2. Data collection   

For subnanometre-resolution analysis of coats by STA, imaging should be performed on a high-end cryo-microscope with a stable stage, equipped with a direct detector and an energy filter (Dodonova *et al.*, 2017 ▸; Hutchings *et al.*, 2018 ▸; Schur *et al.*, 2016 ▸). Tilt series are typically collected at defoci ranging from −1.5 to −3.5 µm. The transmission of high-frequency signal degrades with dose accumulation, limiting the total electron dose used as distributed across the tilt series. Signal is best transmitted at lower tilts, which have a lower apparent thickness and thereby suffer fewer inelastic scattering events. An efficient, dose-symmetric tilt scheme which maximizes the amount of electron dose spent on lower tilt images has been established: the first image is collected at zero tilt, and then the sample is tilted, alternating both directions at incremental angles (Hagen *et al.*, 2017 ▸). A tilt angle range of ±60° with 3° increments and exposures of up to 150 e^−^ Å^−2^ is typically used (Hagen *et al.*, 2017 ▸; Wan *et al.*, 2017 ▸; Schur *et al.*, 2016 ▸; Bharat *et al.*, 2017 ▸). Multiple frames can be collected per tilt and aligned using the *MotionCor*2 software (Zheng *et al.*, 2017 ▸).

To achieve subnanometre resolutions, a high number of subtomograms must be available, which necessitates high-throughput automated data collection. For example, to analyse the COPII budding reconstitution described above, *SerialEM* software was used to automatically collect 90 tomograms (Mastronarde, 2005 ▸).

### 2.3. Pre-processing and reconstruction   

Accurate tilt series alignment is central to retaining high-resolution information in the reconstructed tomogram. The low electron doses per tilt result in an inherently low signal-to-noise ratio per tilt (SNR), which can affect tilt series alignment by cross-correlation. High-contrast gold fiducial markers are typically added to *in vitro* reconstitution reactions and are used to manually minimize the alignment residual error between tilts, which can be performed using the *ETomo IMOD* interface.

The incremental electron damage suffered by the sample results in the progressive loss of higher frequency information. The contribution of this low-SNR information can be reduced by dose compensation, which attunes progressively lower frequency components with the accumulation of electron dose (Grant & Grigorieff, 2015 ▸). Current tomography software that implements dose compensation includes *emClarity* and *IMOD* (Himes & Zhang, 2018 ▸; Mastronarde & Held, 2017 ▸).

Contrast transfer function (CTF) correction is essential for the recovery of higher resolution information. Appropriate CTF correction relies on accurate defocus determination. Modern CTF detection software such as *CTFFIND*4 (Rohou & Grigorieff, 2015 ▸) and *Gctf* (Zhang, 2016 ▸) is usually able to detect CTF oscillations seen in Thon rings for all direct detector images across a tilt series. However, the accuracy can significantly decrease for the higher tilts, where less signal is available to guide CTF estimation, owing to increased thickness and the defocus gradient. The consequences of this are partially attenuated in the process of dose compensation. The resolution of STA is maximized if CTF correction is performed using defocus values adjusted across the thickness of the reconstruction (Jensen & Kornberg, 2000 ▸). This has recently been implemented in the *NovaCTF* 3D CTF correction software, in which CTF correction is performed during the tomogram reconstruction process (Turoňová *et al.*, 2017 ▸; Himes & Zhang, 2018 ▸; Kunz & Frangakis, 2017 ▸).

To reconstruct the original 3D density from its 2D projections (*i.e.* the tilt series), the finely aligned and pre-processed tilt series are back-projected from their assigned orientation angles. To gain contrast for better visualization in the initial phases of processing, filters can be applied during the reconstruction process. For initial tomogram visualization, weighted back-projection (WBP) with iterations of simultaneous iterative reconstruction technique (SIRT)-like filtering can be performed using the *Etomo IMOD* interface (Mastronarde & Held, 2017 ▸).

### 2.4. Subtomogram extraction and averaging   

Subtomogram particle picking of repetitive coat subunits assembled on membranes is facilitated by *a priori* information about the expected localization and orientation of coat subunits and their distribution. Typically, subtomograms are extracted using centre coordinates generated on the surface of the coated membranes. These can be defined upon manual membrane segmentation (Castaño-Díez, 2017 ▸) or generated automatically using tubular or spherical geometrical parameters (Zanetti *et al.*, 2013 ▸; Faini *et al.*, 2012 ▸). For repetitive assemblies, coat lattices are typically oversampled, and the convergent alignment of subtomograms containing redundant information is used to define the centre of unique particles (Zanetti *et al.*, 2013 ▸). Initial rough assignment of two Euler angles is possible for coats, as subunits are all expected to be oriented normally to the membrane. In the case of tubules, in-plane rotations can also be assigned relative to the direction of the tube axis. This both helps to obtain an initial reference for alignments, as discussed below, and limits the angular search to small ranges, significantly decreasing the computational time. Initial alignments are often performed using binned 8× data, and low-resolution signal can be further enhanced by applying SIRT-like filters. Subtomogram averaging can be performed using a number of software packages, including *Dynamo*, *emClarity*, *TOM*/*AV*3, *PEET*, *Jsubtomo*, *MLTOMO*, *RELION*, *PyTom* and *EMAN*2 (Castaño-Díez, 2017 ▸; Himes & Zhang, 2018 ▸; Förster *et al.*, 2005 ▸; Nicastro *et al.*, 2006 ▸; Heumann *et al.*, 2011 ▸; Huiskonen *et al.*, 2010 ▸, 2014 ▸; Stölken *et al.*, 2011 ▸; Scheres, 2012 ▸; Hrabe *et al.*, 2012 ▸; Galaz-Montoya *et al.*, 2015 ▸, 2016 ▸; Bharat *et al.*, 2015 ▸).

Initial references are typically generated by averaging extracted subtomograms and smoothening (*i.e.* low-pass filtering) the result to obtain a featureless membrane density. This is used for initial alignment of the subtomograms, which helps to define particle centres and can often constitute a first cleaning step. The subtomogram dataset is typically split in half and processed separately during further alignment, following a ‘gold-standard’ approach (Scheres & Chen, 2012 ▸). Starting with subtomograms that are binned 8× and heavily low-pass filtered, binning factors and low-pass filtering can be gradually decreased while restricting the angular search in order to reveal higher resolution information without falling into local minima (Hutchings *et al.*, 2018 ▸; Zanetti *et al.*, 2013 ▸; Faini *et al.*, 2012 ▸; Dodonova *et al.*, 2015 ▸, 2017 ▸). It is important that conservative low-pass filters are applied at each iteration in order to avoid bias from the high-resolution noise contained in the reference. Resolution can be estimated using Fourier shell correlation (FSC) between the two half datasets (Harauz & van Heel, 1986 ▸). The two half datasets are combined for the final reconstruction and low-pass filtered to the resolution indicated by the FSC at the 0.143 threshold (Rosenthal & Henderson, 2003 ▸).

## 3. Studying coat protein assemblies by tomography   

Coat complexes assembled on their cognate membranes form complex and unique 3D objects. Since their global assembly underlies their function, it is informative to study their structure within the context of the coat in the act of remodelling membranes. Cryo-ET is suited for investigating interactions between coat protein subunits in their assembled state and between coat proteins and the membrane. Within the 3D volume of the tomogram, coat proteins at different heights along the microscope axis, which overlap in 2D projection images, are resolved, allowing their isolation and analysis *in silico*. Averaging multiple copies of identical building blocks within an object of interest through STA leads to higher resolution views of coat subunits. Available structural information from X-ray crystallography and single-particle studies can be fitted into densities determined by STA. Together with the ability to map the relative positions and orientations of the aligned coat components within each tomogram, this provides information about the structure of subunits, their assembled architecture, and in some cases their physiological context, providing a pseudo-atomic model of unique pleiomorphic coat assemblies where the quality of the fits permits.

**Figure 5:**
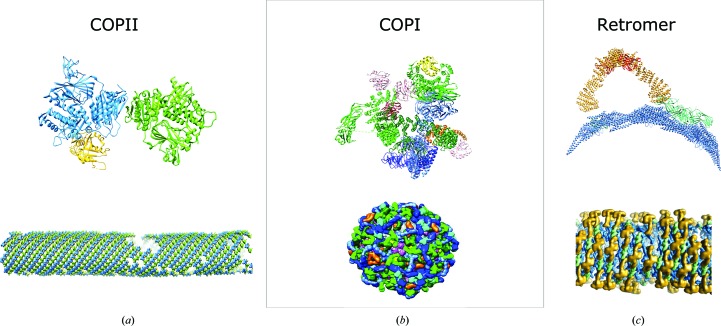
Structural insights gained from the study of coat proteins. The top panels represent fitted structural models of asymmetric units. The bottom panels show the global coat arrangement. (*a*) Inner COPII coat (Hutchings *et al.*, 2018 ▸; PDB entry 6gni). Colour scheme: Sec23, blue; Sec24, green; Sar1, yellow. (*b*) COPI (Dodonova *et al.*, 2015 ▸, 2017 ▸; PDB entry 5nzr). Colour scheme: Arf1, pink; γ-COP, light green; β-COP, dark green; ζ-COP, yellow; δ-COP, orange; β′-COP, light blue; α-COP, dark blue. (*c*) Retromer (Kovtun *et al.*, 2018 ▸; PDB entry 6h7w). Colour scheme: Vps5, blue; Vps29, red; Vps35, yellow; Vps26, green. The images are reproduced with permission from AAAS and Springer Nature.

High-resolution structures of coat proteins have elucidated essential protein–protein and protein–membrane interactions that underpin coat assembly on membranes (Hutchings *et al.*, 2018 ▸; Kovtun *et al.*, 2018 ▸; Dodonova *et al.*, 2017 ▸). These recent studies shed light on the structural basis of the flexibility that allows the formation of carriers of different shapes and sizes to accommodate the needs of the cell. We outline the insights gained in recent studies of COPII, COPI and retromer (Fig. 5 ▸).

### 3.1. The structure of COPII assembled *in vitro*   

**Figure 6:**
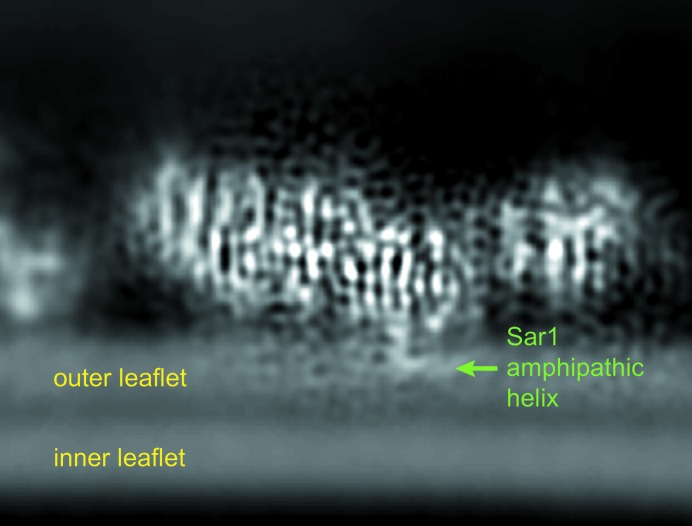
Association of the Sar1 GTPase with the ER membrane as seen in EMDB entry EMD-0044 (Hutchings *et al.*, 2018 ▸). Sar1 inserts its N-­terminal helix into the membrane using a helical hook bent by 90°.

Our recent cryo-ET and STA studies of *in vitro* reconstituted COPII budding yielded a structure of the COPII inner coat, composed of Sar1 and Sec23/24, at an average resolution of 4.9 Å, providing important new insights into the mechanisms of coat assembly and membrane remodelling (Fig. 5 ▸ *a*). Firstly, it revealed the association of the coat and the Sar1 GTPase with the membrane. We observed for the first time how the N-terminal amphipathic helix of Sar1 is associated with the membrane: a helical hook is bent by 90° and inserts horizontally into the outer leaflet, running roughly parallel to the tube axis (Fig. 6 ▸). Also, the membrane-proximal surface of the Sec23/24 dimer is tightly associated with the bilayer, in particular a negatively charged patch on the surface of Sec24, which corresponds to its zinc-finger domain. Together, amphipathic helix insertion and the scaffolding action of the concave inner surface of Sec23/24 provide the force necessary to remodel membranes into tubules (Hutchings *et al.*, 2018 ▸).

Moreover, our structure unveiled how the inner and outer coat are associated: through the binding of motifs embedded within the outer coat disordered C-terminal tails to two distinct sites on Sec23. Together with previous biochemical and X-ray crystallographic studies (Antonny *et al.*, 2001 ▸; Bi *et al.*, 2007 ▸; Ma & Goldberg, 2016 ▸), our work revealed how the outer coat affects the stability of COPII assemblies and the formation of the inner coat lattice and suggested how this process might be regulated during COPII-mediated budding.

### 3.2. The structures of COPI and retromer *in vitro* and *in situ*   

Two other coat protein assemblies, COPI and retromer, were analysed by cryo-ET and STA of *in vitro* reconstituted assemblies and within the cell. Three consecutive studies have investigated the structure of COPI assembled *in vitro* from purified coatomer components on GUVs (Faini *et al.*, 2012 ▸; Dodonova *et al.*, 2015 ▸, 2017 ▸). The most recent study determined the structure of the COPI leaf, composed of a coatomer complex bound to two Arf1 GTPases, to 9.2 Å resolution (Fig. 5 ▸ *b*; Dodonova *et al.*, 2017 ▸). This showed an asymmetric subunit of a coatomer component bound to two membrane-interacting Arf1 molecules and elucidated interactions with ArfGAP2, which is essential for GTP hydrolysis by the membrane-bound Arf1 GTPase. Recent developments in cellular preparation with focused ion beam (FIB) milling allowed the study of protein assemblies in their native intracellular context by cryo-ET. This enabled the identification of novel interactions, the proteomic context and intracellular localization. Since coat proteins interact with diverse cargoes in a native intracellular context, *in situ* structural studies can reveal the interactions of coat proteins with cargo molecules. The power of *in situ* studies has been demonstrated in a recent study of COPI as a way of identifying protein–protein interactions that are relevant in an intracellular context (Bykov *et al.*, 2017 ▸). Here, the authors demonstrated that coat architecture *in vitro* mimics that seen in physiological contexts. Moreover, they identified a novel density that is not present in *in vitro* reconstitutions, which was attributed to cargo or cargo receptors as informed by previous biochemical knowledge regarding the cargo-binding site of β-COPI.

The structure of retromer as assembled *in vitro* from purified retromer Vps5 components on liposomes was recently determined to subnanometre resolution (Fig. 5 ▸ *c*; Kovtun *et al.*, 2018 ▸). This revealed a membrane-coating array of the bar-domain protein Vps5, from which arches formed by retromer components Vps26, Vps35 and Vps29 protruded. Vps26 acted as an adaptor, while Vps29 was exposed at the tip of the arches, allowing modulation of the assembly by cytosolic factors. When subtomograms were positioned into the original tomogram density with their respective orientations, a semi-regular helical array was observed. Similarly to COPI, *in situ* validation of the structure of retromer obtained from *in vitro* reconstitutions confirmed that *in vitro* reconstituted assemblies mirrored native retromer arrangements within the cell (Kovtun *et al.*, 2018 ▸).

## 4. Challenges and future perspectives   

Cryo-ET and STA have been applied to the study of units that do not form arrays, such as bacterial secretion systems (Chang *et al.*, 2016 ▸; Nans *et al.*, 2015 ▸; Hu *et al.*, 2015 ▸, 2017 ▸; Wang *et al.*, 2017 ▸; Chang *et al.*, 2018 ▸), ribosomes (Pfeffer, Burbaum *et al.*, 2015 ▸; Khoshouei *et al.*, 2017 ▸; Englmeier *et al.*, 2017 ▸; Pfeffer, Woellhaf *et al.*, 2015 ▸; Pfeffer *et al.*, 2012 ▸; Ortiz *et al.*, 2010 ▸), the proteasome (Asano *et al.*, 2015 ▸; Albert *et al.*, 2017 ▸) and transmembrane proteins (Kudryashev *et al.*, 2016 ▸; Davies *et al.*, 2012 ▸).

While some studies have demonstrated that near-atomic resolution is possible with STA (Schur *et al.*, 2016 ▸), technical and practical challenges limit tomographic analysis of many targets. Practically, data collection for tomography is low-throughput, as a tomogram takes significant time to be collected, and often the number of particles to average per tomogram is low. Coat protein assemblies are good systems as they form orderly arrays of repeating subunits and often contain hundreds of subunits per tomogram (Zanetti *et al.*, 2013 ▸; Dodonova *et al.*, 2017 ▸; Kovtun *et al.*, 2018 ▸), but for many other samples, especially when targeted within cellular environments, achieving large datasets remains a challenge (Asano *et al.*, 2015 ▸; Albert *et al.*, 2017 ▸; Chang *et al.*, 2016 ▸). To minimize the time needed for tomogram collection, a fast-incremental tilt method has recently been proposed, which could hold the potential to optimize data collection for high-resolution analysis (Chreifi *et al.*, 2019 ▸).

Data processing also requires significant user expertise and manual intervention. The ongoing developments in automatic tilt series alignment procedures and the establishment of user-friendly software for STA will greatly benefit the speed of processing (Mastronarde & Held, 2017 ▸; Chen *et al.*, 2017 ▸; Castaño-Díez, 2017 ▸; Himes & Zhang, 2018 ▸; Noble & Stagg, 2015 ▸).

*In vitro* reconstitutions provide control over sample composition and enable studies of the effect of varying the reaction environment in a controlled manner. Additionally, investigating the effect of mutations, which is central to the validation of structural information, is technically straightforward and is isolated from possible off-target effects. However, isolation from potential interacting partners, especially transient interactors that have not been biochemically established, limits discovery and the collection of exhaustive structural data. Information is also lost regarding the cellular context of the complex of interest. *In situ* studies can bridge the gap between *in vitro* analysis with purified proteins and the cell, allowing the identification of novel interacting surfaces, validating *in vitro* structural information, and providing global spatiotemporal insight (Kovtun *et al.*, 2018 ▸; Bykov *et al.*, 2017 ▸; Mahamid *et al.*, 2016 ▸; Englmeier *et al.*, 2017 ▸). While the challenges faced by *in situ* structural determination are beyond the scope of this review, we note that automated and sophisticated technologies for identifying assemblies of interest through template-matching may allow higher throughput and lower subjectivity (Lučič *et al.*, 2013 ▸; Asano *et al.*, 2015 ▸).
